# A New Equation to Estimate Peripherally Inserted Central Catheter Length

**DOI:** 10.3390/medicina60030417

**Published:** 2024-02-29

**Authors:** Hosu Kim, Soo-Buem Cho, Sung-Eun Park, Sa-Hong Jo, Sung-Gong Lim, Yujin Jeong, Jung-Ho Won, Won-Jeong Yang, Ho-Cheol Choi, Jong-Hwa Ahn, In-Chul Nam

**Affiliations:** 1 Department of Internal Medicine, Changwon Fatima Hospital, Changwon 51394, Republic of Korea; narulake@naver.com; 2 Department of Radiology, College of Medicine, Ewha Womans University, Seoul 07804, Republic of Korea; kingnose80@gmail.com; 3 Department of Radiology, Gyeongsang National University School of Medicine and Gyeongsang National University Changwon Hospital, Changwon 51472, Republic of Korea; jo452y@naver.com (S.-H.J.); limsg2002@gnuh.co.kr (S.-G.L.); yjj3290@gnuh.co.kr (Y.J.); 4 Department of Radiology, Gyeongsang National University School of Medicine and Gyeongsang National University Hospital, Jinju 52727, Republic of Korea; circlehoya@gnuh.co.kr (J.-H.W.); yangwonjeong@gnuh.co.kr (W.-J.Y.); hocheol72@gnuh.co.kr (H.-C.C.); 5 Department of Internal Medicine, Gyeongsang National University School of Medicine and Gyeongsang National University Changwon Hospital, Changwon 51472, Republic of Korea; jonghwaahn@gnuh.co.kr; 6 Department of Radiology, Jeju National University School of Medicine, Jeju Natuional University Hospital, Jeju 63241, Republic of Korea; sky_hall@naver.com

**Keywords:** intensive care unit, elbow crease to the cavoatrial junction length, equation, peripherally inserted central catheter, sex, weight

## Abstract

*Background and Objectives*: Peripherally inserted central catheter (PICC) placement plays an important role in clinical practice. This study aimed to provide an equation for the proper estimation of catheter length in cases of PICC placement without imaging guidance in relation to patient height, weight, sex, and age. *Materials and Methods*: For 1137 PICC placement cases in both arm veins of 954 patients at a single center, the elbow crease to the cavoatrial junction length (ECL) was calculated as follows: ECL = (PICC length) + (distance from the elbow crease to the puncture site). We analyzed the relationship between ECL and patient characteristics and suggested a new equation for ECL based on height, weight, sex, and age. *Results*: The average ECL was 48.0 ± 2.4 cm in the right side and 51.0 ± 3.0 cm in the left side. ECL in the right arm was significantly correlated with patient height, sex, and age, whereas the ECL in the left arm was additionally significantly correlated with patient weight. The ECL (cm) prediction model was as follows: right ECL = 26.32 + 1.33 × (female = 1, male = 2) − 0.02 × age (years) + 0.13 × height (cm); left ECL = 22.09 + 1.28 × (female = 1, male = 2) + 0.02 × age (years) + 0.14 × height (cm) + 0.042 × weight (kg). *Conclusions*: The appropriate PICC length was predicted based on the patient’s height, weight, sex, and age. The equations in our study can help predict the optimal catheter length and can be automatically calculated using computerized patient information for bedside procedures in PICC.

## 1. Introduction

Since the first attempt to enter upper extremity veins to access the central venous system in 1912, peripherally inserted central catheters (PICCs) offer intermediate-term to long-term venous access and are a safe and convenient way to administer various medications, such as parenteral nutrition and antibiotics [1]. It has become an important component of patient management and its use has increased in recent years.

Obtaining the correct position of the catheter tip is a critical aim for central venous catheter placement. The National Association of Vascular Access Networks and Infusion Nurses Society recommends that the catheter tip be located in the lower one-third of the superior vena cava (SVC), close to the junction of the SVC and right atrium [2,3]. The malpositioning of central venous catheters has been associated with delayed line use, increased costs, and increased rates of complications, including arrhythmia, phlebitis, and thrombosis.

Patients in an intensive care unit (ICU) are required to maintain stable and long-term venous catheters. Although there are currently no data on how many PICCs are performed without fluoroscopic guidance, PICCs are often inserted at the patient’s bedside without fluoroscopic guidance. This is because the roles and environments of the medical personnel in each country and hospital are diverse. In our institution, approximately 20% of PICCs are inserted without fluoroscopic guidance in an ICU.

After catheter placement, the tip position is confirmed using portable digital radiography [1]. Other methods to confirm the tip position utilize electrocardiography gating or newer devices such as the electromagnetic positioning system [4]. However, the bedside placement of PICCs without such devices is common in many institutions because of cost-effectiveness. Although there are a few reports about the length of insertion guidelines for PICCs in relation to height, these studies provided a formula for predicting only the distance from the elbow crease to the carina and were conducted either on the right or left arm [5,6].

This single-center study aimed to predict the appropriate catheter length by considering patient characteristics, such as height, weight, age, and sex, before the PICC procedure through both upper arm veins, which are particularly useful in cases of non-fluoroscopic PICC insertion. This single-center study provided an equation for the proper estimation of catheter length in cases of PICC placement without imaging guidance.

## 2. Materials and Methods

### 2.1. Study Population

We retrospectively reviewed the clinical records of 1039 consecutive adult patients who underwent PICC placement at the Gyeongsang National University Changwon Hospital between April 2017 and May 2021. This study was approved by the Institutional Review Board of Gyeongsang National University Changwon Hospital (IRB No. GNUCH 2021-05-020) and was performed in accordance with the committee guidelines. Data collection was only conducted from 25 June 2021 to 26 July 2021 and we only used de-identified data collected during clinical practice. The requirement for informed consent was waived because of the retrospective study design and anonymization of personal information.

All cases were treated indifferently because the opposite arm or different veins were selected at each time point of the repeat procedures in the same patient because of local infection and possible thrombosis or venous stenosis associated with the previous PICC procedure. Information on the approached veins is summarized in Table 1. Data regarding patient age, sex, weight, and height were collected from hospital electronic medical records.

The exclusion criteria were incomplete records of height or weight, incomplete insertions of PICC owing to central vein stenosis or occlusion, and severe mediastinal shifts due to pneumonectomy or atelectasis.

### 2.2. PICC Procedure Technical Details

All PICC insertions were performed in the intervention suite, with the patient in the supine position. The arm to be initially accessed was determined after considering the following factors: (1) arm dominance, (2) patient or clinician preference, (3) history of previous PICC insertion, (4) history of previous procedural difficulty or failure of PICC insertion, and (5) any signs of infection or swelling around the puncture site on the arm. Ultrasonography (US) was performed to determine whether the veins were accessible; however, the catheter-to-vein ratio was not measured. The elbow joint was fully extended, and the arm was externally rotated as much as possible and abducted to approximately 60°. Usable basilic or brachial veins were selected for US examination. Vein puncture was performed approximately 5–15 cm above the elbow crease using a micropuncture needle in the PICC set (5 Fr. Dual lumen Pro-PICC CT Basic IR set, Medcomp, Harleysville, PA, USA or 5 Fr. Dual lumen Xcela Power injectable PICC, Navilyst Medical, Marlborough, MA, USA). After the insertion of the calibrated guidewire with the tip at the level of the cavoatrial junction and dilatation of the tract with the dilator sheath, the catheter was cut according to the length estimated by the calibrated guidewire inside the body (from the puncture site to the level of the cavoatrial junction). The cavoatrial junction was assumed to be 2.4 vertebral body units below the carina on fluoroscopy, and the PICC tip was placed in this position [7]. The PICC was inserted along the guidewire, and the external portal of the catheter was fixed to the skin using a fixation device in the PICC set.

### 2.3. Measurement Methods and Analyses

**Figure 1:**
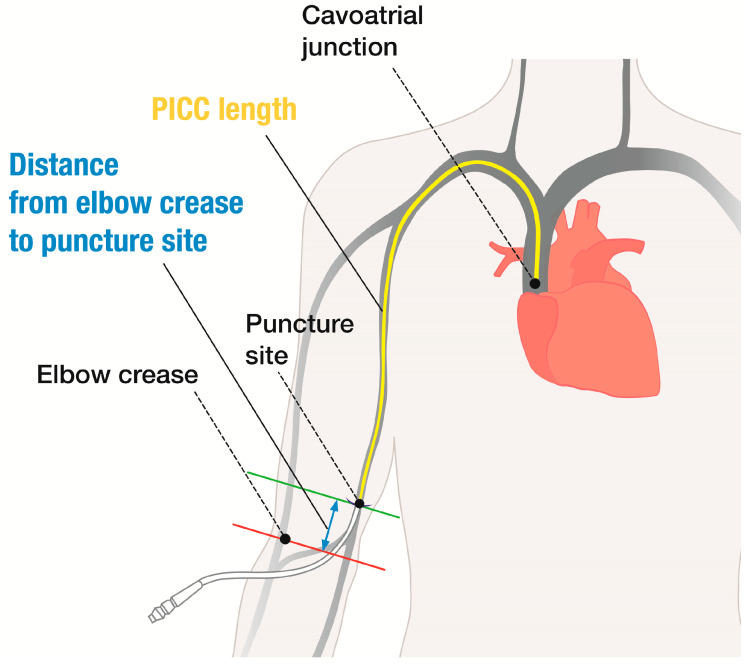
Elbow crease to the cavoatrial junction length. ECL = PICC length + distance from the elbow crease to the puncture site. ECL, elbow crease to the cavoatrial junction length; PICC, peripherally inserted central catheter. ECL, the elbow crease to the cavoatrial junction length.

The PICC length (PCL) inside the body and the distance from the elbow crease to the puncture site of the skin (DEP) were recorded for all procedures. Based on the records of PCL and DEP, we calculated the length of the upper extremity vein from the elbow crease to the cavoatrial junction (ECL; ECL = PCL + DEP) (Figure 1). To evaluate the factors related to ECL, variables such as age, sex, height (cm), weight (kg), PCL, and DEP were reviewed. The equation for ECL through both upper arm veins, in relation to patient characteristics, was obtained from these results.

Statistical analyses were performed using the SPSS software version 24 (IBM Corp., Armonk, NY, USA). Continuous data are expressed as mean ± standard deviation, whereas categorical data are expressed as percentages or absolute numbers. The chi-squared test was used for categorical data; the *t*-test was used for continuous data. A correlation analysis was performed using Pearson’s correlation coefficient to analyze correlations between continuous variables. Linear regression analyses were used to analyze the relationship between the upper extremity vein length and other related variables and to obtain a predictive model. An analysis of variance was conducted if there was a significant difference of ECL among the groups of punctured veins, although the number of patients in whom the cephalic vein was punctured was small. The results are expressed as *β* ± standard error for linear regression. Statistical significance was set at *p* < 0.05.

## 3. Results

### 3.1. Participants

In total, 123 cases were excluded (62 incomplete records of patient height or weight, 47 incomplete insertions of PICC owing to central vein stenosis or occlusion, and 14 severe mediastinal shifts owing to pneumonectomy or atelectasis), and 1137 cases in 954 patients constituted the study group. In 129 of the 954 patients, PICC procedures were performed more than twice (mean, 2.3 cases; range, 2–7 cases).

### 3.2. Participant Baseline Characteristics

A total of 1137 cases were included in this study. The mean age was 68.6 ± 14.7 years (range, 15–96 years). In this study, 627 cases (55.1%) were male patients. The mean height of the patients was 161.9 ± 9.6 cm (range, 132–187 cm) and the average weight was 61.7 ± 24.9 kg (27.8–83.8 kg). PICC placement was performed in 266 cases (23.4%) on the right arm and in 871 cases (76.6%) on the left arm. Among the PICCs on the right side, 177 (15.6%) were placed on the basilic vein, 87 (7.7%) on the brachial artery, and 2 (0.2%) on the cephalic vein. On the left arm, 507 procedures (44.6%) were performed on the basilic vein, 360 (31.7%) on the brachial vein, and 4 (0.4%) on the cephalic vein. The procedure took 6.7 ± 3.4 min (range 3–30 min) on average. Immediate complications occurred in three patients (three cases of hematoma).

### 3.3. Differences in Variables between Males and Females

There were significant differences in baseline characteristics between males and females. Among the enrolled patients, male patients were younger than female patients (66.1 ± 14.2 for males, 71.6 ± 14.8 for females, *p* < 0.001). Moreover, males had a higher height (168.0 ± 6.9 for males, 154.3 ± 6.7 for females, *p* < 0.001) and weight (65.7 ± 15.5 for males, 55.4 ± 11.8 for females, *p* < 0.001) than females. Males had more puncture sites on the right side (26.6% for males, 19.8% for females, *p* = 0.007) than females. Catheter lengths, including ECL, PCL, and DEP, were longer in males than in females on both the left and right sides. However, there was no significant difference in procedure time between males and females (6.6 ± 3.4 for males, 6.9 ± 3.3 for females, *p* = 0.123).

### 3.4. Catheter Length Analysis

The average PCL was 38.8 ± 3.5 cm on the right side and 42.1 ± 3.7 cm on the left side. The mean ECL was 48.0 ± 2.4 cm on the right side and 51.0 ± 3.0 cm on the left side. When the correlation analysis was performed with other variables, age, height, and weight were statistically correlated with PCL and ECL on both the left and right sides. Younger age, male sex, and greater height and weight were correlated with longer PCL and ECL. The data are presented in Table 2.

### 3.5. Catheter Length Prediction Model Analysis

**Figure 2:**
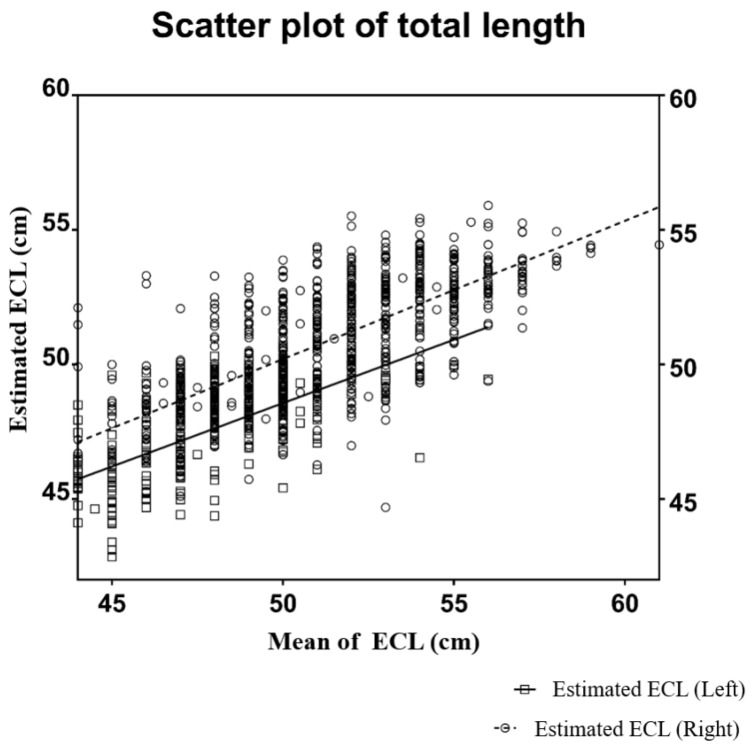
Scatter plot of the elbow crease to the cavoatrial junction length. Right elbow crease to the cavoatrial junction length (ECL) (cm) = 26.318 + 1.332 × (female = 1, male = 2) − 0.018 × age (years) + 0.128 × height (cm); left ECL (cm) = 22.085 + 1.278 × (female = 1, male = 2) + 0.020 × age (years) + 0.143 × height (cm) + 0.042 × weight (kg).

An ECL predictive model was obtained through a linear regression analysis using variables such as age, sex, height, and weight. Age, sex, and height were significantly correlated with the right side ECL. The right ECL prediction model was as follows: right ECL (cm) = 26.318 + 1.332 × (female = 1, male = 2) − 0.018 × age (years) + 0.128 × height (cm), R^2^ = 0.466. Based on the results of the linear regression analysis, the variables that correlated with the left ECL were age, sex, height, and weight, and the predictive model was as follows: left ECL (cm) = 22.085 + 1.278 × (female = 1, male = 2) + 0.020 × age (years) + 0.143 × height (cm) + 0.042 × weight (kg), R^2^ = 0.515 (Figure 2).

## 4. Discussion

The optimal position of the central venous catheter tip is important and requires careful consideration of catheter type, insertion site, and patient habitus. Catheter tip malpositioning can lead to complications. When the catheter tip is placed too high, there is an increased risk of catheter malfunction, thrombosis, infection, and SVC injury. However, when the catheter tip is placed too low, there may be an increased risk of arrhythmia, pericardial placement, and tamponade, which are some of the most serious complications [8]. ICU patients, in particular, may be more susceptible to arrhythmias [9,10]. Even if the catheter length is appropriate, arrhythmia can occur with just the guide wire during the procedure. Knowing the appropriate length of the catheter before the procedure can prevent the occurrence of such cardiac arrhythmias by inserting a guide wire of the appropriate length before inserting the catheter.

The cavoatrial junction cannot be reliably identified on plain chest radiography or fluoroscopy, although it is generally accepted that it is always located below the carina [11]. A recent study suggested that the position of the central catheter tip in relation to the carina could be described using the thoracic vertebra as an internal ruler and that the position of the cavoatrial junction in adults was reliably estimated to be 2.4 vertebral body units below the carina [7]. In our study, we considered 2.4 vertebral body units below the carina as a radiologic landmark for the PICC tip position.

**Figure 3:**
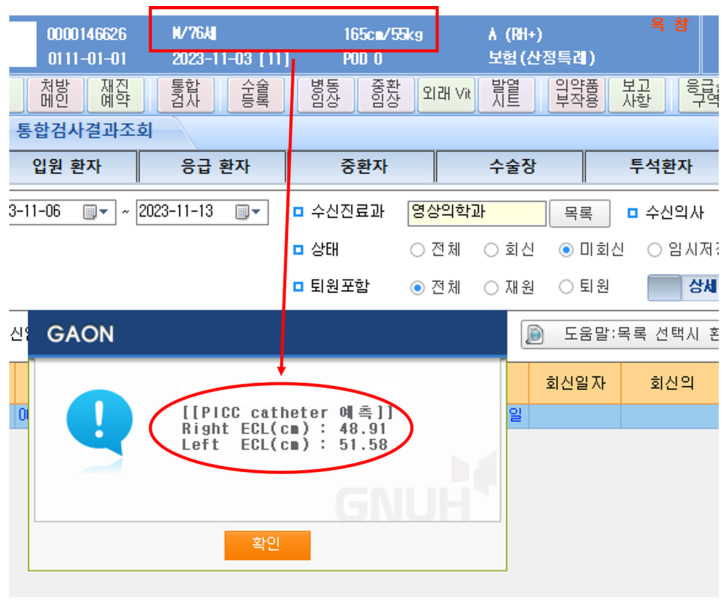
Example of automatic elbow crease to the cavoatrial junction length. Presentation based on patient characteristics. Using the elbow crease to the cavoatrial junction length (ECL) prediction equation, entered into the hospital computer program, the estimated ECL (ellipse box) is automatically presented based on the patient’s characteristics (square box) in the electronic chart. The Korean word “예측” means prediction.

For PICC implantation in the ICU, postprocedural chest radiography is the most effective method to confirm the appropriate positioning of the catheter tip. However, ICU patients are critically ill and often require frequent radiography scans and nearby patients and staff may be at risk of radiation exposure if appropriate protective measures are not taken during portable radiography. The amount of radiation exposure varies depending on factors such as distance, radiation intensity, location of the body part being imaged, and imaging technique [12]. If the PCL suitable for a patient can be predicted before the procedure, portable radiography need not be performed. The equation can also be used for PICC insertion using the blind pushing technique [13]. Based on the formula obtained in our study, a computerized request was made to the hospital to automatically present the ECL according to the patient’s characteristics and access arm in the patient’s electronic chart (Figure 3). Therefore, we were able to reduce unnecessary chest radiography scans by having the operator know the automatically calculated, expected ECL before PICC insertion and determine the PCL accordingly.

Various maneuvers, such as digital pressure over the ipsilateral supraclavicular fossa and head turning toward the side of cannulation, have been recommended to improve the success rate of blind PICC insertion [14]. When a bedside PICC is inserted, its tip may be incorrectly positioned in the ipsilateral internal jugular vein. In such cases, US examination of the ipsilateral neck may be helpful [15]. Additionally, blind PICC insertion does not have a higher risk of upper extremity deep vein thrombosis or other complications than conventional PICC insertion [13,16]. Previous studies have investigated formulas for predicting PCL in blind PICCs [5,6]. These studies provided a formula for predicting only the distance from the elbow crease to the carina, were conducted only on the right arm or the left arm, and included <150 patients. However, the formula derived in our study can calculate the distance from the elbow crease to the cavoatrial junction, allowing for a more accurate length prediction than that of previous studies.

Although there are studies about the length of the upper extremity veins including basilic, cephalic, axillary, subclavian, and innominate veins, as well as SVC, those are only for the average size adult [6]. So, we consider that our equation is more useful because it reflects the patient height, weight, sex, and age to a statistical significance. Although the estimation from the equation is not used initially during the brachial implantation of the venous port, we believe that this equation will be helpful for the calculation of the length of the upper arm vein to the cavoatrial junction in the case of exchange.

Our study had several limitations. First, it was performed at a single institution. Second, proper estimation of the catheter length did not indicate the proper position of the tip, and this study had several biases because all patients were in the supine position with the abducted arm position. Third, the reason body weight did not act as a variable in the right ECL prediction equation, unlike that in the left, has not been elucidated. Finally, there may be racial differences in the length of the extremity [17]; however, this could not be determined in our study because all of our patients were Asian. A multicenter, large-scale study should be conducted to obtain scientific evidence for this and other populations.

## 5. Conclusions

The length derived from the new equation based on patient age, sex, height, and weight could be used as a guideline for the insertion length of bedside PICCs. An automatic calculation program that uses a patient’s electronic medical record system can be used for convenience. If the national health information system is used in the future, a more accurate equation will be able to be deduced.

## Figures and Tables

**Table 1:** Selected veins in male and female patients.

	Basilic	Cephalic	Brachial	Total
Males	374	3	250	627
Females	310	3	197	510
Total	684	6	447	1137

**Table 2:** Relationship between catheter length and patient characteristics.

	PCL		ECL	
	r	*p*-Value	r	*p*-Value
Age	−0.298	<0.001	−0.331	<0.001
Males	39.6 ± 3.5	<0.001	49.2 ± 2.1	<0.001
Females	37.6 ± 3.4	<0.001	46.3 ± 1.9	<0.001
Height	0.397	<0.001	0.646	<0.001
Weight	0.326	<0.001	0.372	<0.001

Values are shown as mean ± standard deviation; data are expressed as r (correlation coefficient). PCL, peripherally inserted central catheter length; ECL, the elbow crease to the cavoatrial junction length. *p* < 0.05.

## Data Availability

All relevant data are contained within the paper.
